# BAG6 deficiency induces mis‐distribution of mitochondrial clusters under depolarization

**DOI:** 10.1002/2211-5463.12677

**Published:** 2019-06-04

**Authors:** Mizuki Hayashishita, Hiroyuki Kawahara, Naoto Yokota

**Affiliations:** ^1^ Laboratory of Cell Biology and Biochemistry, Department of Biological Sciences Tokyo Metropolitan University Japan

**Keywords:** BAG6, CCCP, mitochondria, p62, Parkin, PINK1

## Abstract

Accumulation of damaged mitochondria is implicated in a number of neurodegenerative disorders, including Parkinson's disease. Therefore, the machinery for mitochondrial quality control is important for the prevention of such diseases. It has been reported that Parkin‐ and p62/sequestosome 1 (SQSTM1)‐mediated clustering and subsequent elimination of damaged mitochondria (termed mitophagy) are critical for maintaining the quality of mitochondria under stress induced by uncoupling agents such as carbonyl cyanide *m*‐chlorophenyl hydrazone. However, the molecular mechanisms underlying mitochondrial translocation to the perinuclear region during mitophagy have not been adequately addressed to date. In this study, we found that BCL2‐associated athanogene 6 (BAG6; also known as BAT3 or Scythe) is required for this process. Indeed, RNA interference‐mediated depletion of endogenous BAG6 prevented Parkin‐dependent relocalization of mitochondrial clusters to the perinuclear cytoplasmic region, whereas BAG6 knockdown did not affect the translocation of Parkin and p62/SQSTM1 to the depolarized mitochondria and subsequent aggregation. These results suggest that BAG6 is essential for cytoplasmic redistribution, but not for clustering, of damaged mitochondria.

**Abbreviations**
BAG6: BCL2‐associated athanogene 6
CCCP: carbonyl cyanide *m*‐chlorophenyl hydrazone
DMSO: dimethyl sulfoxide
EGTA: ethylene glycol tetraacetic acid
ER: endoplasmic reticulum
ERAD: endoplasmic reticulum‐associated degradation
MAM: mitochondria‐associated ER membranes
MFN: mitofusin
PBS: phosphate buffered saline
PINK1: PTEN‐induced kinase
siRNA: small interference RNA
SQSTM1: sequestosome 1
TA: tail‐anchored
TOMM20: translocase of outer mitochondrial membrane 20
UBX: ubiquitin regulatory X
VCP: valosin‐containing protein

Mitochondria are dynamic organelles that continually undergo repeated cycles of fission, fusion, and redistribution inside cells 1. Mitochondria are responsible for essential cellular functions such as energy production, calcium homeostasis, amino acid metabolism, and programmed cell death 2. Dysfunction of mitochondria has been implicated in a number of diseases, including Parkinson's disease 3, 4, 5, and the machinery for mitochondrial quality control to prevent accumulation of damaged mitochondria has been studied 6, 7. Parkin, a parkinsonism‐linked E3 ubiquitin ligase, is essential for such quality control in response to loss of the mitochondrial membrane potential in a PTEN‐induced kinase 1 (PINK1)‐dependent manner 8, 9, 10, 11, 12. Indeed, both Parkin and ubiquitin phosphorylated by PINK1 are recruited to the surface of depolarized mitochondria when cells are treated with carbonyl cyanide *m*‐chlorophenyl hydrazone (CCCP), a mitochondrial uncoupling agent 13, 14, 15. Parkin mediates polyubiquitination of mitochondrial substrates 14, 16, and these modifications recruit the LC3‐binding adaptor protein p62/sequestosome 1 (SQSTM1) to damaged mitochondria 12. Subsequently, p62/SQSTM1 mediates the aggregation/clustering of dysfunctional mitochondria through polymerization via its PB1 domain 12, 17. It was reported that Parkin also contributes to change in the distribution of damaged mitochondria in a minimal area within the cell for sequestration 3. Since mitochondria also sustain damage with aging 5, the Parkin/PINK1‐ and p62‐mediated mitochondrial quality control pathway is of importance to avoid a number of age‐related neurodegenerative diseases 5, 18, 19. Although the machinery responsible for quality control of damaged mitochondria has been studied extensively, the molecular mechanism underlying the control of mitochondrial translocation during depolarization has only recently received attention.

Accumulating evidence suggests that substrates for both the co‐ and post‐translational endoplasmic reticulum (ER)‐targeting pathways are eliminated in a BCL2‐associated athanogene 6 (BAG6)‐dependent manner when their ER targeting fails 20, 21, 22, 23, 24, 25. BAG6 is a hydrophobicity‐oriented chaperone/holdase 26, 27, 28 and has been shown to be a receptor for tail‐anchored (TA) protein biogenesis 29, 30, 31 and for targeted degradation of newly synthesized defective polypeptides that expose aberrant hydrophobicity in the cytosol 32, 33, 34. Thus, these reports shed light on the crucial role of BAG6 in the quality control of newly synthesized and mislocalized proteins 35, 36, 37, 38, 39. Recent mass spectrometry‐based comprehensive human protein–protein interaction network databases have suggested potential interactions between BAG6 and several mitochondrial outer membrane proteins, such as translocase of outer mitochondrial membrane 20 (TOMM20) 40, 41. Furthermore, BAG6 was recently identified as mitofusin (MFN) 1/2‐interacting protein and plays a role in the ubiquitin‐dependent turnover of MFNs in the absence of mitochondrial fission 42. These results suggest that BAG6 might be a candidate regulator of mitochondrial quality control. However, critical functions of BAG6 in the mitochondria have not been adequately investigated, and further studies are awaited.

In this study, we examined the impact of BAG6 knockdown on the CCCP‐induced distribution of mitochondria. Whereas prominent mitochondrial clusters at the perinuclear region were clearly observed after CCCP treatment in control small interfering RNA (siRNA)‐transfected cells, RNA interference‐mediated depletion of endogenous BAG6 prevented Parkin‐dependent translocation of depolarized mitochondria. We also found that BAG6 was localized on the surface of mitochondria regardless of the presence of CCCP. These results demonstrate that BAG6 might have a critical role in the cytoplasmic redistribution of damaged mitochondria in the Parkin‐mediated mitophagy pathway.

## Materials and methods

### Mammalian cell culture and transfection

HeLa cells were cultured in Dulbecco's modified Eagle's medium (Wako Pure Chemical Industries, Ltd., Osaka, Japan) supplemented with 10% heat‐inactivated calf serum at 37 °C under 5% CO_2_ atmosphere. Transfections of the expression vectors were performed with polyethyleneimine ‘MAX’ transfection reagent (Polysciences, Inc., Warrington, PA, USA) or Hily Max (DOJINDO, Kumamoto, Japan) according to the protocols supplied by the manufacturers. In the case of CCCP treatment, the cells were exposed to 20 μm CCCP (Wako Pure Chemical Industries, Ltd.) starting at 24 h after cDNA transfection and continuing for the indicated time.

### Subcellular fractionation

HeLa cells (2.5 × 10^6^) were harvested, washed with PBS, and resuspended in hypotonic buffer (225 mm mannitol, 75 mm sucrose, 0.1 mm EGTA, 30 mm Tris/HCl, pH 7.4). The cells were homogenized with 15 strokes in a Dounce homogenizer. The homogenate was transferred to a 2‐mL tube and centrifuged for 5 min at 600 ***g***. The resultant supernatant was centrifuged for 10 min at 7000 ***g*** to separate the crude mitochondrial fraction (ppt.) from the cytosolic fraction (sup.). A microsomal fraction was separated from the cytosolic fraction by centrifugation for 1 h at 100 000 ***g***. Mitochondria‐associated ER membranes (MAM) and the pure mitochondrial fractions were isolated from the crude mitochondrial fraction by Percoll gradient fractionation according to a protocol described previously 43. Briefly, the crude mitochondrial fraction was resuspended in 340 μL of mitochondria resuspending buffer (225 mm mannitol, 5 mm HEPES‐KOH, pH 7.2, 0.5 mm EGTA) and overlaid onto 1.3 mL of Percoll medium (225 mm mannitol, 25 mm HEPES‐KOH, pH 7.2, 1 mm EGTA, 30% Percoll). After addition of 400 μL of mitochondria resuspending buffer onto the crude mitochondrial fraction, the tube was centrifuged for 30 min at 95 000 ***g***. The pure mitochondria layer was collected and used succeeding experiments after resuspended in mitochondria resuspending buffer.

### RNA interference

BAG6 depletion in human cells was performed as described previously 24, 33 with a duplex siRNA covering the target sequences; 5′‐UUUCUCCAAGAGCAGUUUAtt‐3′ (*BAG6* siRNA #1) and 5′‐CAGAAUGGGUCCCUAUUAUtt‐3′ (*BAG6* siRNA #3). MISSION siRNA universal negative control 1 (Sigma Genosys, Tokyo, Japan) was used as a negative control 44. The transfection of duplex siRNA was performed using Lipofectamine 2000 (Thermo Fischer Scientific, Waltham, MA, USA) for HeLa cells, according to the manufacturer's protocol. The efficacy of knockdown was verified by western blotting.

### Immunological analysis

For western blot analyses, samples were subjected to SDS/PAGE and transferred onto polyvinylidene difluoride membranes (Merck Millipore, Burlington, MA, USA). The membranes were blocked with PBS containing 5% skim milk and 0.1% Tween‐20, and then immunoblotted with indicated antibodies, followed by detection with ECL Western Blotting Detection Reagents (GE Healthcare, Chicago, IL, USA), Clarity™ Western ECL substrate (Bio‐RAD, Hercules, CA, USA), or Immobilon™ Western Chemiluminescent HRP substrate (Merck Millipore). The following antibodies were used in this study: anti‐BAG6 32, anti‐Flag M2 monoclonal F3165 (Sigma), anti‐T7 69522 (Merck Millipore), anti‐TOMM20 sc‐11415 (Santa Cruz Biotech, Dallas, TX, USA), anti‐calnexin C4731 (Sigma), anti‐tubulin T9026 (Santa Cruz Biotech), and horseradish peroxidase‐conjugated antibody against mouse or rabbit immunoglobulins (GE Healthcare).

### Microscopic observations

For immunocytochemical observations, HeLa cells were grown on coverslips and were washed twice with PBS and fixed with 4% paraformaldehyde in PBS for 15 min at room temperature. Permeabilization was carried out in a solution containing digitonin (50 µg·mL^−1^) for 5 min at 37 °C. The cells were blocked with 3% bovine serum albumin in PBS for 30 min at room temperature and then reacted with a series of primary antibodies at room temperature for 1 h or overnight at 4 °C, followed by 1‐h incubation with secondary antibodies at room temperature. Alexa Fluor® 488‐conjugated anti‐mouse IgG and Alexa Fluor® 594‐conjugated anti‐rabbit IgG antibodies (Thermo Fischer Scientific) were used as secondary antibodies. To observe the nucleus, we stained cells with Hoechst‐33342 (DOJINDO). Immunofluorescent images were obtained using laser scanning confocal microscopy system LSM710 (Carl Zeiss, Oberkochen, Germany).

### Plasmid construction

The full‐length cDNA for Parkin of *Homo sapiens* (GenBank accession numbers: 169790968) was amplified by PCR from HEK293 cDNA library. The PCR products were cloned into pCI‐neo‐based mammalian expression vector (Promega, Madison, WI, USA) with an N‐terminal 3×Flag or 3×T7 ‐tags sequence. The expression vectors were used for experiments after verification of the inserted sequence. All experiments were performed in accordance with ethical guidelines in Tokyo Metropolitan University, and the licensing committee approved the experiments.

## Results

### BAG6 plays an important role in the perinuclear localization of mitochondria under depolarizing condition

**Figure 1:**
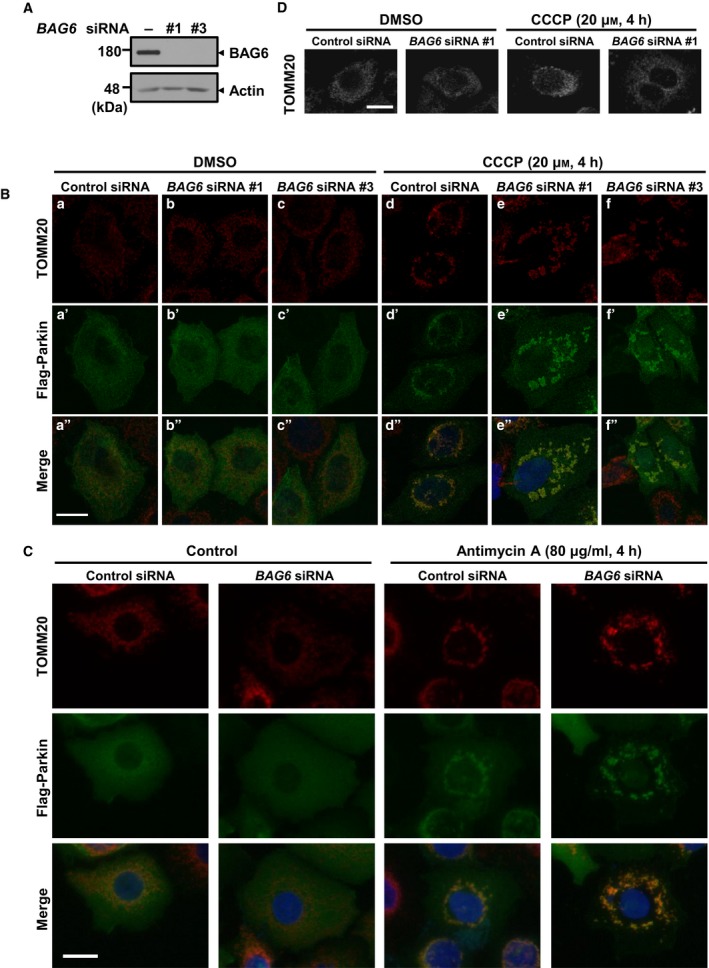
BAG6 knockdown disrupts translocation of mitochondria to the perinuclear region under depolarization. (A) Efficacy of BAG6 knockdown by siRNA in HeLa cells. HeLa cells were transfected with control or *BAG6* siRNA. Three days after transfection, the expression of BAG6 protein was examined by western blotting. (B) Three days after transfection with siRNA, Flag‐Parkin‐transfected HeLa cells were treated with CCCP for 4 h and stained with anti‐TOMM20 (mitochondria; red), anti‐Flag (Parkin; green), and Hoechst (nucleus; blue). Scale bar represents 20 μm. (C) HeLa cells were transfected with Flag‐Parkin together with control or *BAG6* siRNA #1, followed by treatment of 80 μg·mL^−1^ antimycin A for 4 h. Cells were stained with anti‐TOMM20 (mitochondria; red), anti‐Flag (Parkin; green), and Hoechst (nucleus; blue). Scale bar represents 20 μm. (D) Immunocytochemistry with TOMM20 of BAG6 knockdown and control HeLa cells without ectopic expression of Parkin. Cells were treated with 20 μm CCCP for 4 h or DMSO (negative control). Scale bar represents 20 μm.

BAG6 has been shown to play roles in many biological processes, such as TA protein targeting to the ER 31, misfolded/mislocalized protein degradation 21, 45, transcriptional control through regulating histone demethylation 46, and endoplasmic reticulum‐associated degradation (ERAD) 27. Because most of the reported BAG6 functions are restricted to the cytoplasm and the nucleus, we examined whether BAG6 functions in other cellular compartments. In a *in silico* survey using an interactome database, we identified several mitochondrial proteins such as TOMM20 as BAG6‐interacting proteins; thus, here we aimed to investigate the role of BAG6 in mitochondria 40, 41. Furthermore, mitochondrial fusion proteins MFN1/2 are reported to interact with BAG6 42. To examine the roles of BAG6 in mitochondria, we knocked down the *BAG6* gene with siRNA in HeLa cells. The efficacy of the knockdown was confirmed by western blotting with a specific antibody (Fig. 1A). Under non‐stressed conditions, we did not see any significant differences in the distribution and amounts of mitochondria in BAG6‐knockdown cells compared with control cells (Fig. 1Ba,b). These results suggest that BAG6 is not essential for the maintenance of mitochondrial dynamics under basal conditions, which is consistent with previous observations that BAG6 perturbation showed an effect only in *DRP1*‐knockout cells 42.

**Figure 2:**
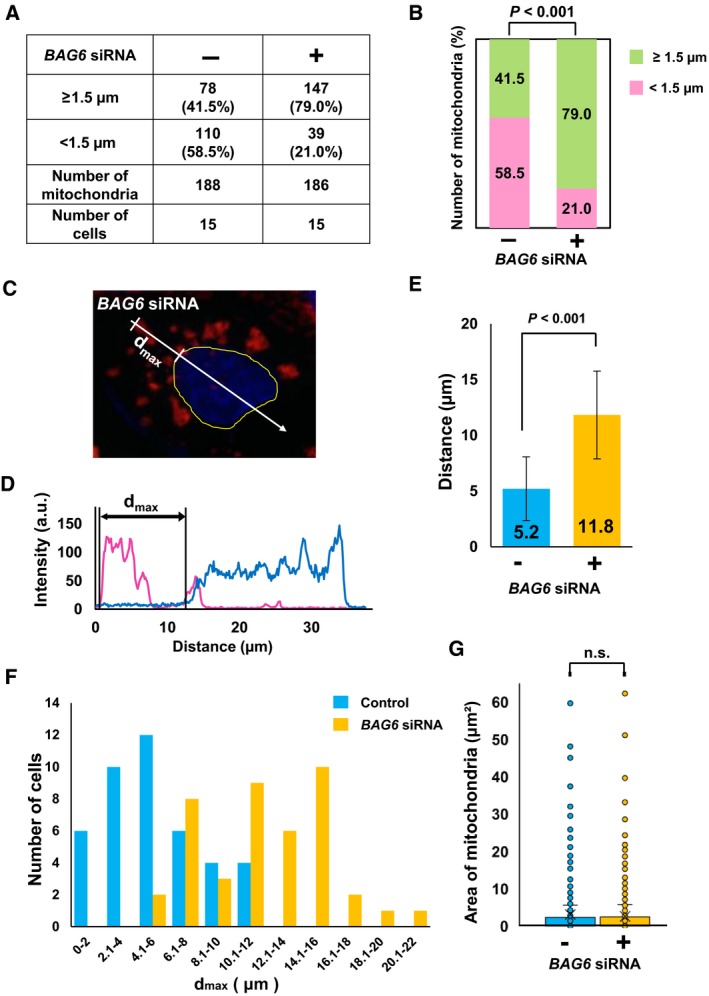
Effects of *BAG6* siRNA on distribution and size of mitochondria in Flag‐Parkin‐transfected cells. (A) HeLa cells were transfected with Flag‐Parkin, together with control or *BAG6* siRNA. The distance of each mitochondrion from the nucleus was measured. The numbers of mitochondria located < 1.5 and ≥ 1.5 μm from the edge of the nucleus were counted. We selected 15 control and 15 BAG6‐knockdown cells and measured 188 and 186 randomly selected mitochondria, respectively. (B) Data shown in (A) are presented as a bar graph. Chi‐squared test was used for statistical analysis. (C–F) HeLa cells were transfected with Flag‐Parkin and non‐targeting or *BAG6* siRNA. Cells were treated with 20 μm CCCP for 4 h. Fluorescence intensities along the line shown in C are displayed in D. Magenta and blue lines indicate TOMM20 and Hoechst signals, respectively. *d*_max_ is the maximum distance between the nucleus and the furthest mitochondrial cluster in each cell. (E) Average *d*_max_ of knockdown and control cells [mean ± standard deviation (SD); *n* = 42]. Bars represent the mean ± SD (Student's *t*‐test). (F) Distributions of *d*_max_ of control cells (blue) and BAG6‐knockdown cells (orange) are presented in a bar graph. (G) Box‐and‐whisker plots showing the area of mitochondria from control and BAG6‐knockdown cells. n.s.: not significant.

Mitochondria are highly dynamic organelles that undergo fission and fusion according to cellular status, and their shape, quality, and quantity are precisely regulated 2. Mitochondria can be damaged by reactive oxygen or other toxic molecules, resulting in the loss of mitochondrial membrane potential. Such damaged mitochondria are removed by selective mitochondrial autophagy (also termed mitophagy) 6, 7. Since BAG6‐interacting proteins such as valosin‐containing protein (VCP) are essential for mitophagy 47, we hypothesized that BAG6 might be involved in the quality control of mitochondria under conditions of stress. To test this hypothesis, we used the uncoupling reagent CCCP to induce mitochondrial depolarization to produce damaged mitochondria. Addition of CCCP would induce depolarization of the mitochondrial membrane potential and result in recruitment of PINK1 and Parkin to the mitochondrial surface for induction of mitophagy 17. Because HeLa cells express very limited endogenous Parkin, which is essential for the pathway 48, we transiently expressed Flag‐tagged Parkin following knockdown experiments. The Parkin‐transfected cells showed significant changes in cellular distribution of mitochondria with marked puncta/clusters within 4 h after the addition of CCCP, and they translocated around the nucleus (Fig. 1Bd,d″), while mitochondria were distributed uniformly in the cytoplasm and showed a fibrillar structure before CCCP treatment, which is consistent with previous observations 12, 17. We repeatedly observed that most of the mitochondria in BAG6‐knockdown cells formed clusters after CCCP treatment, but failed to translocate to the area close to the nucleus and were scattered within the cytoplasm (Fig. 1Be,e″). To exclude the possible off‐target effects of siRNA, we performed independent knockdown experiments with different siRNA duplex (Fig. 1A). We confirmed that the essentially identical impairment of the perinuclear localization of damaged mitochondria was observed by the treatment of *BAG6* siRNA #3 (Fig. 1Bf,f”). We also examined the effects of the different inhibitor of cellular respiration, antimycin A, to disrupt proton gradient in the mitochondria. After 4 h of antimycin A treatment, control cells showed perinuclear clustering of mitochondria as described previously 49. In BAG6‐knockdown cells, impairment of perinuclear clustering of damaged mitochondria was obvious (Fig. 1C). These observations suggest that the phenotype of BAG6 knockdown on damaged mitochondria with antimycin A was similar, if not identical, to those with CCCP. It is noteworthy that Parkin expression was necessary to induce significant differences in the distribution of mitochondria under depolarization between control and BAG6‐knockdown cells (Fig. 1D), suggesting that impairment of perinuclear localization occurs in the Parkin‐mediated pathway. This observation was confirmed by counting the number of mitochondria within 1.5 μm from the nucleus (Fig. 2A,B). In control cells, 58.5% of the mitochondria were translocated within 1.5 μm from the nucleus, while only 21.0% of mitochondria were located in the perinuclear region in BAG6‐knockdown cells. In addition, when we compared the distance of the furthest mitochondria from the nucleus, there was a significant difference between control and BAG6‐knockdown cells (Fig. 2C–F), confirming that clustered mitochondria failed to translocate to the perinuclear region in BAG6‐compromised cells.

It has been reported that relocation of Parkin to the mitochondrial surface results in ubiquitination of mitochondrial outer membrane proteins 10. This ubiquitination induces the recruitment of p62/SQSTM1 through its PB1 domain to the mitochondria, which is essential for the aggregation of mitochondria but not for mitophagy 12, 17. We found that BAG6 perturbation did not affect the translocation of Parkin and p62/SQSTM1 to the mitochondrial surface after CCCP treatment (Figs 1Be′,f′ and 3), suggesting that BAG6 acts downstream of p62/SQSTM1 in the Parkin‐mediated mitophagy pathway. To examine whether BAG6 knockdown affects the clustering of mitochondria under depolarization, we measured the size of each mitochondrion in the control and BAG6‐knockdown cells. The average sizes of the clusters in control and BAG6‐knockdown cells were 3.07 and 3.14 μm^2^, respectively; this difference was not statistically significant (Fig. 2G). Next, we examined whether BAG6 knockdown affects the clearance of mitochondria after depolarization. In control siRNA‐treated cells, the amount of TOMM20 decreased in a time‐dependent manner after CCCP treatment. We found that the CCCP‐induced elimination of mitochondrial protein was observed in the BAG6‐knockdown cells as fast as control siRNA‐treated cells. This observation suggested that BAG6 is dispensable for the clearance of mitochondria under depolarizing conditions (Fig. 4). Collectively, these findings suggest that BAG6 is critical for translocation, but neither for clustering nor clearance, of mitochondria to the perinuclear region after mitochondrial aggregation mediated by p62/SQSTM1.

**Figure 3:**
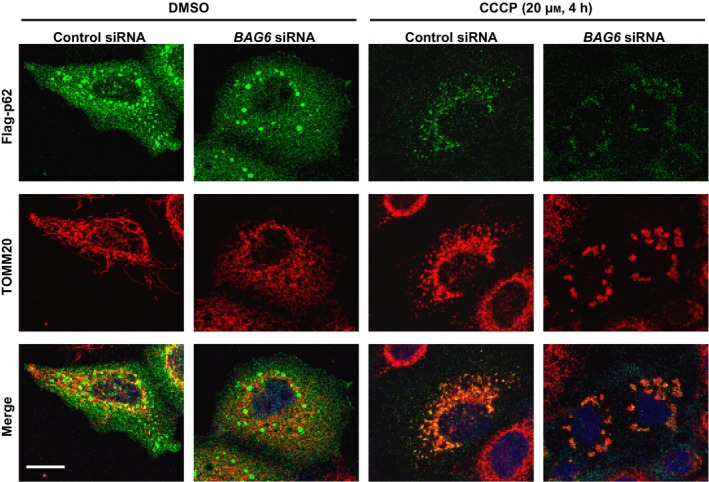
BAG6 knockdown does not impair relocalization of p62/SQSTM1 to depolarized mitochondria. HeLa cells were transfected with siRNA and T7‐Parkin. After 4 h of CCCP treatment, the cells were immunostained for Flag‐p62/SQSTM1. Scale bar represents 20 μm.

**Figure 4:**
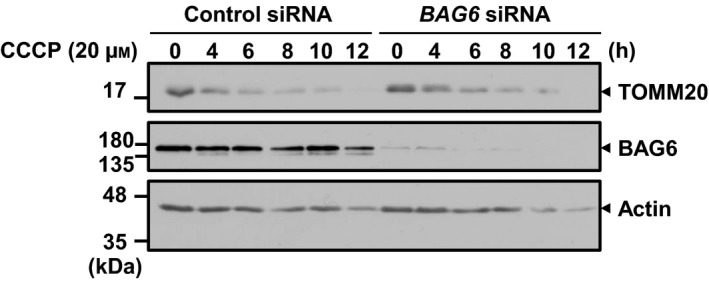
BAG6 knockdown did not affect the mitochondrial clearance rate induced by CCCP. HA‐Parkin‐expressing cells were treated with or without *BAG6* siRNA #1 and incubated with 20 μm of CCCP. Cells were harvested at the indicated time points and were subjected to SDS/PAGE followed by immunoblotting with antibodies for BAG6, actin, and TOMM20.

### BAG6 localizes in a pure mitochondrial fraction

**Figure 5:**
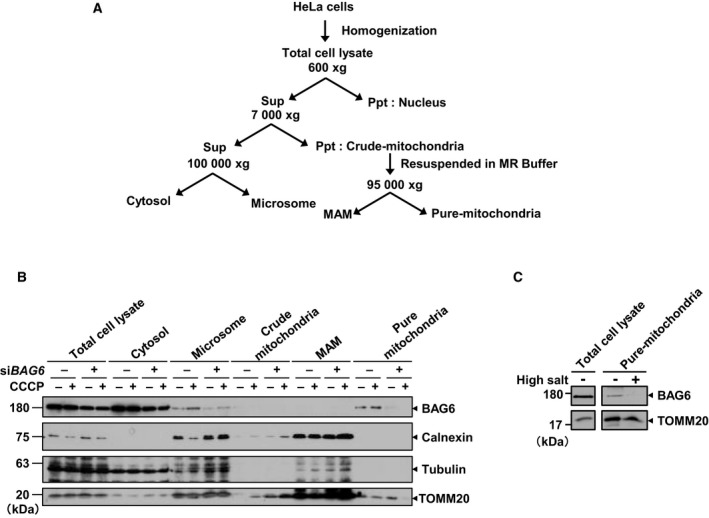
BAG6 was localized in the mitochondrial fraction. (A) Schematic diagram of the fractionation protocol. (B) Fractions were analyzed by western blotting. Tubulin, calnexin, and TOMM20 were used as markers for the cytoplasm, microsome, and mitochondria, respectively. The grouping of blots cropped from different parts of the same membrane. (C) Pure mitochondrial fractions isolated from HeLa cells were incubated with high‐salt buffer 1 m KCl, 600 mm mannitol, 20 mm HEPES‐KOH, pH 7.5, 2 mm MgCl_2_, and 1 mm PMSF) for 15 min on ice and then centrifuged for 5 min at 10 000 ***g***. The resultant high‐salt‐washed pure mitochondria were recovered as a pellet. The samples were separated by sodium dodecyl sulfate‐polyacrylamide gel electrophoresis and immunoblotted for BAG6 and TOMM20, which was used as a control for mitochondria‐embedded protein.

To obtain further insight into the role of BAG6 in mitochondria, we examined whether BAG6 is localized in mitochondria in the presence or absence of CCCP. We fractionated HeLa cells into cytosolic, microsomal, MAM, and pure mitochondrial fractions by Percoll‐density ultracentrifugation 43 (Fig. 5A), and BAG6 or marker proteins were detected using western blotting. The purity of each isolated fraction was confirmed by the presence or absence of the following marker proteins: calnexin for ER, tubulin for cytosol, and TOMM20 for mitochondria. BAG6 was mainly localized in the cytosolic and microsomal fractions, which was in accordance with previous observations that BAG6 is involved in the protein triage system in the cytosol 45. Interestingly, the BAG6 signal was found in the pure mitochondrial fraction and the signal disappeared on BAG6 depletion (Fig. 5B). This experiment also suggested that BAG6 is localized in mitochondria regardless of the presence of CCCP (Fig. 5B). Since BAG6 possesses neither a transmembrane domain nor mitochondrial targeting signal, we next examined how BAG6 associates with mitochondria. For this purpose, the pure mitochondrial fraction was washed with high‐salt buffer. We found that BAG6 disappeared from the mitochondrial fraction after washing with high‐salt buffer (Fig. 5C), whereas TOMM20, which is known to be embedded in the mitochondrial outer membrane, remained. This result suggests that BAG6 is not embedded or incorporated into the mitochondrial outer membrane. On the basis of these data, we suggest that BAG6 localizes on the surface of mitochondria. MFN1/2 and TOMM20 could have a role in tethering of BAG6 on mitochondria, although further detailed analysis is required for future study.

## Discussion

Quality control of mitochondria is critical for preventing neurodegenerative disorders, and its impairment has been reported to contribute to the onset of Parkinson's disease 3, 4, 5. Parkin‐mediated mitophagy is an adapted mechanism that contributes to the selective elimination of damaged mitochondria, and dysfunction of the pathway leads to pathogenesis 3, 4, 5, 6, 7. Under depolarizing condition induced by a decoupling agent, PINK1 is stabilized and recruits phosphorylated Parkin to the mitochondrial surface 13, 14, 15. Following ubiquitination of outer mitochondrial membrane proteins by Parkin, clustering of depolarized mitochondria is promoted by p62/SQSTM1, and mitochondria are translocated subsequently toward the perinuclear region 12, 17.

Despite recent progress in understanding the machinery for quality control of damaged mitochondria, it is currently unclear how damaged mitochondrial translocation is regulated during depolarization. Here, we show that BAG6 is involved in perinuclear localization of mitochondria, suggesting that BAG6 is a novel regulator of the mitochondrial quality control system and may be involved in retrograde transport induced by Parkin.

It has been reported that polyubiquitinated aggregation‐prone proteins are transported along microtubules toward the microtubule‐organizing center to form a perinuclear inclusion structure termed the aggresome, which reduces the toxicity of aggregation‐prone proteins 50. Given that p62/SQSTM1 is a component of the aggresome and involved in aggresome formation 51, and that BAG6 localized in the aggresome and its knockdown suppressed the formation of aggresomes in HeLa cells 32, it is plausible that BAG6 might sequester damaged mitochondria to reduce their toxicity by limiting the spread of reactive oxygen species as is observed in the aggresome formation 49. Furthermore, it has been reported that HDAC6 is responsible for mitochondrial aggregation as well as aggresome formation in concert with dynein motors 52. Since BAG6 was also reported to be involved in these processes (32, this study), it is plausible that BAG6 might collaborate with HDAC6 during the course of perinuclear clustering of mitochondria.

Although Parkin is required for BAG6‐dependent perinuclear localization of mitochondria (Fig. 1B–D), we were not able to assess the relationship between BAG6 and p62/SQSTM1. Considering that perinuclear mitochondrial clustering has also been reported in hypoxic conditions and the process is also controlled by the microtubule and dynein‐dependent pathway 53, we suggest that association between p62/SQSTM1 and the mitochondrial surface might be necessary but not sufficient for BAG6‐dependent translocation. It is worth noting that hypoxia also triggers BCL2‐interacting protein 3‐dependent mitophagy through hypoxia‐inducible factor 1, and this pathway is distinct from Parkin‐mediated mitophagy 18. Although we have focused on the phenotype of BAG6 knockdown exclusively in Parkin‐mediated mitophagy, it would be interesting to study a role of BAG6 in other mitophagy pathways to understand the general role of BAG6 in the perinuclear localization of damaged mitochondria.

We found that BAG6 is not restricted to the cytosol and ER, but is also localized in mitochondria (Fig. 4B). However, the functional relationship between mitochondrial BAG6 and its role in the perinuclear localization of damaged mitochondria remains unclear. Therefore, what is the functional role of BAG6 on the mitochondrial surface? It has been reported that VCP, in concert with ubiquitin regulatory X (UBX) domain‐containing protein 1, is recruited to the mitochondrial surface and assists degradation of MFN1/2, which promotes degradation of damaged mitochondria 54. VCP reportedly interacts with UBX domain‐containing protein 8, another UBX protein, as well as BAG6 to escort ERAD substrates to the proteasome 27, 55. Considering that BAG6 is an important functional protein that associates with VCP and UBX proteins 53, BAG6 might act with these proteins to aid efficient degradation of mitochondrial protein during Parkin‐mediated mitophagy.

In addition, impaired signal recognition particle function results in detrimental targeting of ER transmembrane proteins to mitochondria, which leads to mitochondrial defects 56. Similarly, the loss of GET3, the yeast homologue of TRC40/ASNA1, induces mistargeting of some types of TA proteins to mitochondria 57. Based on these observations, we speculate that BAG6 also plays a pivotal role in the triage of defective or mistargeted mitochondrial proteins as has been proposed in the cytosol and on the ER surface 21, 22, 34, 37, 45. Further studies are warranted to clarify the detailed function of BAG6 on the mitochondrial surface.

## Conflict of interest

The authors declare no conflict of interest.

## Author contributions

MH, HK, and NY conceived and designed the experiments. MH performed all the experiments. MH, HK, and NY wrote the paper.
